# Anaerobic Fungi: Past, Present, and Future

**DOI:** 10.3389/fmicb.2020.584893

**Published:** 2020-10-21

**Authors:** Matthias Hess, Shyam S. Paul, Anil K. Puniya, Mark van der Giezen, Claire Shaw, Joan E. Edwards, Kateřina Fliegerová

**Affiliations:** ^1^Systems Microbiology & Natural Product Discovery Laboratory, Department of Animal Science, University of California, Davis, Davis, CA, United States; ^2^Gut Microbiome Lab, ICAR-Directorate of Poultry Research, Indian Council of Agricultural Research, Hyderabad, India; ^3^Anaerobic Microbiology Lab, ICAR-National Dairy Research Institute, Dairy Microbiology Division, ICAR-National Dairy Research Institute, Karnal, India; ^4^Department of Chemistry, Bioscience and Environmental Engineering, University of Stavanger, Stavanger, Norway; ^5^Laboratory of Microbiology, Wageningen University & Research, Wageningen, Netherlands; ^6^Laboratory of Anaerobic Microbiology, Institute of Animal Physiology and Genetics, Czech Academy of Sciences, Prague, Czechia

**Keywords:** anaerobic digestion, carbohydrate-active enzymes, food security, herbivores, methanogenesis, Neocallimastigomycota, rumen, sustainable agriculture

## Abstract

Anaerobic fungi (AF) play an essential role in feed conversion due to their potent fiber degrading enzymes and invasive growth. Much has been learned about this unusual fungal phylum since the paradigm shifting work of Colin Orpin in the 1970s, when he characterized the first AF. Molecular approaches targeting specific phylogenetic marker genes have facilitated taxonomic classification of AF, which had been previously been complicated by the complex life cycles and associated morphologies. Although we now have a much better understanding of their diversity, it is believed that there are still numerous genera of AF that remain to be described in gut ecosystems. Recent marker-gene based studies have shown that fungal diversity in the herbivore gut is much like the bacterial population, driven by host phylogeny, host genetics and diet. Since AF are major contributors to the degradation of plant material ingested by the host animal, it is understandable that there has been great interest in exploring the enzymatic repertoire of these microorganisms in order to establish a better understanding of how AF, and their enzymes, can be used to improve host health and performance, while simultaneously reducing the ecological footprint of the livestock industry. A detailed understanding of AF and their interaction with other gut microbes as well as the host animal is essential, especially when production of affordable high-quality protein and other animal-based products needs to meet the demands of an increasing human population. Such a mechanistic understanding, leading to more sustainable livestock practices, will be possible with recently developed -omics technologies that have already provided first insights into the different contributions of the fungal and bacterial population in the rumen during plant cell wall hydrolysis.

## Introduction

The ability of herbivorous animals to ferment recalcitrant plant fiber into utilizable forms of energy, most cases in the form of volatile fatty acids (VFAs), has been attributed to the trillions of microbial cells that inhabit their gastrointestinal tract (Dearing and Kohl, 2017). Importantly, energy stored within complex plant carbohydrates is made accessible to the host animal only through the synergistic activity of its gut microbes (Morais and Mizrahi, 2019).

Independent of which type of mammalian herbivore digestive physiology is considered, all mammalian herbivores have evolved specialized gut compartments to give home to a complex microbial ecosystem of bacteria, anaerobic fungi (AF), protozoa, methanogenic archaea and bacteriophages (Morgavi et al., 2013). All three of the major types of herbivorous mammals depend on these microbiomes and their proper function to support their health and performance: ruminants (e.g., cattle, goats, and sheep), pseudoruminants (e.g., camelids and hippopotami) and hindgut herbivores (e.g., elephants, donkeys, horses and zebras). Furthermore, in foregut fermenting ruminants and pseudoruminants the forestomach microbes themselves also serve as a substantial source of protein and vitamins for the host unlike in hindgut herbivores (Mizrahi, 2013; Nagaraja, 2016). An excellent review of herbivore gastrointestinal physiology, including detailed drawings, and its role on microbial fermentation of plant biomass was summarized by Dehority (2002).

Foregut fermenters are capable of an enhanced degradation of plant biomass that is facilitated by a prolonged (60–90 h) retention time of the feed material in the rumen, the first and largest of three pre-gastric chambers of the foregut fermenter digestive tract. Gastric digestion in the ruminant digestive system occurs in the abomasum, which is the fourth chamber of the ruminant’s foregut. Plant material in the cecum and colon of a hindgut fermenter, on the other hand, is retained on average only half as long (i.e., 30–40 h in equines) as it is in the ruminant’s digestive tract and is consequently less completely digested (Trinci et al., 1994).

Despite the recent significant advances in our understanding of how bacteria and archaea influence the function, resilience, and the environmental footprint of herbivorous mammals such as ruminants (Kittelmann et al., 2014; Henderson et al., 2015; Liu et al., 2017; Huws et al., 2018; Wallace et al., 2019), our knowledge of AF and their influence on the host animal remains limited. This limited knowledge is perhaps not surprising considering that until the mid-20th century it was still believed that all fungi required oxygen (Trinci et al., 1994). It was only in 1975 that the ground-breaking work of Colin Orpin unequivocally confirmed the existence of AF, changing the accepted dogma of the time. Shortly after AF were first isolated and described in the rumen (Orpin, 1975, 1976, 1977a,b), they were also isolated from the horse cecum (Orpin, 1981). Since then, many more AF have been isolated from a wide range of domesticated and wild herbivores with eighteen different genera characterized to date (Hanafy et al., 2020).

To date, AF have been most extensively studied in ruminants, where they are recognized as an important microbe for good rumen function. This is primarily due to their role as highly efficient degraders of recalcitrant plant material (Li et al., 2017; Gilmore et al., 2019). Rumen AF are also syntrophic partners of the methanogenic archaea (Li et al., 2017; Gilmore et al., 2019). Additional insights into the currently understudied herbivore gut mycobiome has the potential to expand our scientific knowledge about life in the absence of oxygen, as well as open new avenues to improve the feed conversion efficiency of plant-based animal feeds. In this review, we discuss the current understanding of AF biology, ecology and their role in livestock production, along with future perspectives on how their true value can be realized.

## Taxonomy

Anaerobic fungi were first documented over 100 years ago, when their flagellated zoospores were mistakenly identified as flagellate protozoa (Liebetanz, 1910; Braune, 1913). After first being incorrectly classified as Protozoa (Liebetanz, 1910; Braune, 1913), they were reclassified, due to the significant evidence collected over many years by Orpin, as belonging to the fungal phylum Chytridiomycetes (Barr, 1980, 1988). In 2007, they were acknowledged as being a distinct phylum, the Neocallimastigomycota (Hibbett et al., 2007). Recently, Tedersoo et al. (2018) proposed a new fungal subkingdom, Chytridiomycota, grouping the Neocallimastigomycota with two additional phyla, the aerobic Chytridiomycota and Monoblepharomycota, thereby acknowledging the monophyletic origin of the zoosporic chitinous fungi (Ebersberger et al., 2012).

The Neocallimastigomycota contains only one order (*Neocallimastigales*) and one family (*Neocallimastigaceae*) comprising eighteen genera; namely the monocentric rhizoidal *Neocallimastix, Piromyces*, *Oontomyces*, *Buwchfawromyces*, *Pecoramyces*, *Liebetanzomyces*, *Feramyces*, *Agriosomyces*, *Aklioshbomyces*, *Capellomyces*, *Ghazallomyces*, *Joblinomyces*, *Khoyollomyces*, and *Tahromyces*; the polycentric rhizoidal *Anaeromyces* and *Orpinomyces*; and the bulbous *Caecomyces* and *Cyllamyces*. Key morphological features of AF taxa, such as the number of flagella on zoospores, type of thallus and rhizoids, steps of zoosporangial development, and the shape of sporangia, are listed in Table 1.

**TABLE 1:** Key morphological features of characterized genera of anaerobic fungi.

**Genus**	**Morphology [zoospore (z), thallus (t), rhizomycelium (r)]**	**Miscellaneous features**	**References**
*Agriosomyces*	Uniflagellate (z) Monocentric (t) Filamentous (r)	Endogenous and exogenous zoosporangial development, rhizoids are swollen below the sporangial tightly constricted neck, swollen sporangiophores	Hanafy et al. (2020)
*Aklioshbomyces*	Uniflagellate (z) Monocentric (t) Filamentous (r)	Bi or triflagellate zoospores, endogenous and exogenous zoosporangial development, papillated sporangia, pseudo-intercalary endogenous sporangia occasionally, unbranched sporangiophores	Hanafy et al. (2020)
*Anaeromyces*	Uniflagellate (z) Polycentric (t) Filamentous (r)	Sporangia with acuminate (mucronate) apex, can be located on erect, solitary, unbranched sporangiophore, hyphae are highly branched, often with numerous constrictions (sausage-like appearance), sometimes with root-like appearance	Breton et al. (1990)
*Buwchfawromyces*	Uniflagellate (z) Monocentric (t) Filamentous (r)	Extensive rhizoidal system with twisted rhizoids, sporangia with no apical projections, septum can be visible, nuclei located in sporangia, but not observed in sporangiophores or rhizoids	Callaghan et al. (2015)
*Caecomyces*	Uniflagellate (z) Monocentric (t) Bulbous (r)	Bi or quadriflagellate zoospores, vegetative stage is absent of developed branching rhizoidal system, consists of spherical or ovoid bodies (holdfast or haustoria), tubular sporangiophores and bulbous rhizoids, nuclei usually present both in sporangia and vegetative cells	Gold et al. (1988)
*Capellomyces*	Uniflagellate (z) Monocentric (t) Filamentous (r)	Endogenous and exogenous zoosporangial development, unbranched sporangiophores can exhibit subsporangial swelling, zoospores released through apical pore	Hanafy et al. (2020)
*Cyllamyces*	Uniflagellate (z) Polycentric (t) Bulbous (r)	Bi or triflagellate zoospores, bulbous holdfast without rhizoids with multiple sporangia, which can be born on a single elongate or branched sporangiophore, nuclei present in bulbous holdfast and sporangiophores	Ozkose et al. (2001)
*Feramyces*	Polyflagellate (z) Monocentric (t) Filamentous (r)	Extensive highly branched rhizoidal system with wide and narrow hyphae, wide hyphae with constrictions at irregular intervals, single terminal sporangium per thallus with the occasional formation of pseudo-intercalary sporangia, sporangiophores frequently coiled or wide and flattened, often forming an apophysis-like or eggcup-like swelling below the sporangium, both endogenous and exogenous zoosporangial development, zoospores are released through apical pore with the sporangial wall staying intact, or through detachment of the whole sporangium	Hanafy et al. (2018)
*Ghazallomyces*	Polyflagellate (z) Monocentric (t) Filamentous (r)	Endogenous and exogenous zoosporangial development, highly branched rhizoids, unbranched sporangiophores, pleomorphic sporangia with septum, sporangial necks constricted with narrow port, zoospores released through apical pore	Hanafy et al. (2020)
*Joblinomyces*	Uniflagellate (z) Monocentric (t) Filamentous (r)	Biflagellate zoospores, both endogenous and exogenous zoosporangial development, sporangiophores vary in length, zoospores released through wide apical pore resulting in empty cup-shaped sporangium	Hanafy et al. (2020)
*Khoyollomyces*	Uniflagellate (z) Monocentric (t) Filamentous (r)	Endogenous and exogenous zoosporangial development, highly branched rhizoids, intercalary swellings in broad hyphae, multisporangiate thallus, branched sporangiophores with two to four sporangia, zoospores released through wide apical pore	Hanafy et al. (2020)
*Liebetanzomyces*	Uniflagellate (z) Monocentric (t) Filamentous (r)	Endogenous and exogenous zoosporangial development, extensive anucleate rhizoidal system without constrictions, single terminal sporangium per thallus, sporangium with septum on sporangiophore of variable length, sometimes forming eggcup-like structure below the sporangium or showing cyst-like structure. Pleomorphism in sporangial and rhizoidal structures on different substrates is typical	Joshi et al. (2018)
*Neocallimastix*	Polyflagellate (z) Monocentric (t) Filamentous (r)	Rhizoid tubular or inflated below the neck of sporangia, sporangia located on unbranched or branched sporangiophores	Heath et al. (1983)
*Oontomyces*	Uniflagellate (z) Monocentric (t) Filamentous (r)	Intercalary rhizoidal swellings, sporangia never mucronated, formed terminally, long sporangiophores can be separated from the rhizomycelium by distinct constriction	Dagar et al. (2015)
*Orpinomyces*	Polyflagellate (z) Polycentric (t) Filamentous (r)	Polynucleate rhizomycelium of extensively branched hyphae, wider hyphae can have tightly constricted points at close intervals (bead-like or sausage-like appearance)	Barr et al. (1989)
*Pecoramyces*	Uniflagellate (z) Monocentric (t) Filamentous (r)	Biflagellate zoospores, both endogenous and exogenous zoosporangial development, single terminal sporangium formed per thallus, sporangiophores unbranched, often forming apophysis-like or eggcup-like swelling below sporangium. Extensive anucleate rhizoidal system lacks rhizoidal swellings or constrictions	Hanafy et al. (2017)
*Piromyces*	Uniflagellate (z) Monocentric (t) Filamentous (r)	Bi or quadriflagellate zoospores, both endogenous and exogenous zoosporangial development, rhizoids with or without subsporangial swelling, septum often in mature zoosporangia	Barr et al. (1989)
*Tahromyces*	Uniflagellate (z) Monocentric (t) Filamentous (r)	Bi or triflagellate zoospores, both endogenous and exogenous zoosporangial development, branched rhizoids, short swollen sporangiophores, sporangia with septum, sporangial necks constricted	Hanafy et al. (2020)

**FIGURE 1:**
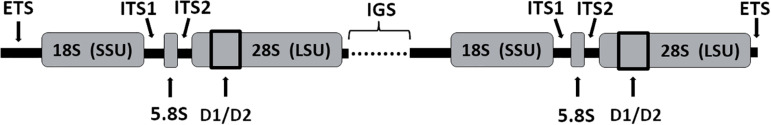
Topology of the ribosomal RNA (*rrn*) operon. Genes of the small (SSU; 18*S*), large (LSU; 28*S*) and 5.8*S* subunit, internal transcribed spacer 1 (ITS1) and 2 (ITS2), flanked by the external transcribed spacer (ETS) regions and linked by the intergenic spacer (IGS).

Although morphological features have been crucial for the classification of AF in the past, this approach is encumbered with difficulties due to the extensive morphological variations, pleomorphism in sporangial and rhizoidal structures, similarities in morphological features of monocentric/uniflagellate genera, failure to produce sporangia, and the absence of zoosporogenesis in some polycentric species. Hence, ribosomal RNA (rRNA) operon-based analysis is also needed to verify and support classification of AF. The topology of the ribosomal RNA (*rrn*) operon, indicating regions that have been used for taxonomic classification, is shown in Figure 1. This culture-independent approach, which in many cases is based on the nucleotide sequence of the internal transcribed spacer 1 (ITS1) region, suggests that the digestive tract of wild and domestic herbivores harbors several clades and genera-equivalent groups within the *Neocallimastigaceae* that have not yet been cultured (Koetschan et al., 2014; Paul et al., 2018).

Although the ITS1 region is currently the molecular marker of choice to assign taxonomy to AF, there are some limitations to this marker, including a high (up to 13%) variation of the ITS1 sequence of clones from a single culture (Callaghan et al., 2015). This variability makes classification of new isolates specifically challenging. Furthermore, the ITS1 region itself ranges in size (Edwards et al., 2008) and recent work of Edwards et al. (2019) indicated preferential amplification of smaller sized ITS1 regions. PCR primer choice is also crucial, which was highlighted by the finding that the sequence amplified by a widely used primer (i.e., MN100F) is not conserved in all AF (Kittelmann et al., 2013; Callaghan et al., 2015).

These problematic aspects of using the ITS1 as phylogenetic marker gene, and the resulting instability of the ITS1 phylogeny, has led to recent efforts to explore the potential of using the sequence of the large 28S rRNA subunit (LSU) as a phylogenetic marker instead (as the 18S rRNA gene is too conserved in AF). It appears that the D1/D2 region of the LSU has a taxonomic resolution similar to the ITS1 region (Wang et al., 2017). However, the more conserved size of the D1/D2 marker and lower heterogeneity within individual cultures enables a more stable phylogenetic backbone for AF classification. Since it provides molecular support for all currently accepted genera of AF, and provides a higher resolution compared to the ITS1 region, utilization of the D1/D2 region of the LSU as a phylogenetic marker seems favorable (Dagar et al., 2011; Wang et al., 2017; Hanafy et al., 2020).

A number of AF specific primers targeting the LSU region have been published (Dollhofer et al., 2016; Nagler et al., 2018), and employed to study AF community composition of environmental samples (Dollhofer et al., 2017; Nagler et al., 2019). However, a sufficiently large reference database for these taxonomic marker genes from previously characterized taxa and their type specimens is still lacking (Wang et al., 2017). There is also a challenge of how to relate LSU sequences to the clades that until now only contain “unculturable” representatives and that were identified by environmentally derived ITS1 sequences (Edwards et al., 2019). Despite the advances of the LSU D1/D2 region as a phylogenetic marker, ITS1 still remains the most accepted phylogenetic marker for AF. As such, it is likely that ITS1 will continue to be used in the near future as the primary barcode to assess AF diversity and community structure in environmental samples. However, it is undisputable that to advance a more accurate and higher resolution classification of members belonging to the AF, a continuous expansion and curation of an AF LSU reference database is need. An alternative option could also be a more complex database that integrates the use of different reference taxonomic marker genes (Wurzbacher et al., 2019). A comprehensive ITS1 sequence database and associated taxonomy files (Koetschan et al., 2014), including the most recently described genera, are available from the AF Network website^1^.

Latest DNA sequence technologies (i.e., PacBio and Nanopore sequencing) generating long reads appear to offer solutions to overcome some of these challenges, specifically the incompleteness, fragmentation and non-overlapping of extant ribosomal data. These new sequencing techniques enable the generation of full-length reference sequences (up to 10 kb in length) that span several regions suitable for taxonomic classification and intraspecies assignment, including the above mentioned ITS1 and LSU gene. Primers targeting the whole fungal ribosomal tandem repeat region, consisting of ETS, SSU, ITS1, 5.8S, ITS2, LSU, and IGS (Figure 1), have already been successfully applied to specimens of three fungal phyla, including early diverging fungi (Wurzbacher et al., 2019). A stable and reliable AF classification system would be enormously facilitated by the ability to widely utilize the complete ribosomal operon sequence as phylogenetic marker, assuming a corresponding well-curated reference database will be available in the future.

## Life Cycle

One of the additional and major challenges that contribute to the current lack of a consistent and standardized taxonomic classification system for AF is the morphological transformations AF undergo throughout their corresponding life cycles (Table 1). AF reproduce asexually and they alternate between a motile zoospore and a non-motile vegetative stage. Flagellate zoospores, which are released from mature sporangia, actively move toward freshly ingested plant tissues in the rumen; a chemotactic response triggered by soluble sugars (Orpin and Bountiff, 1978) and phenolic acids (Wubah and Kim, 1996). Zoospore liberation is influenced by diet, and in ruminants is induced by water-soluble haems and other porphyrins (Orpin and Greenwood, 1986; Orpin, 1994). Although zoospores are motile for several hours (Lowe et al., 1987a), they tend to attach to plant fragments within 30 min after being released from a sporangium (Heath et al., 1986; Edwards et al., 2008). After attachment, the zoospores shed their flagella and form a cyst. The encysted fungus then germinates to produce a fungal thallus that is composed of the sporangium and a filamentous rhizomycelium or a bulbous holdfast.

In most of the monocentric AF, thallus development is of the endogenous type, where on germination, the zoospore cyst develops a germ tube that branches and grows into a rhizoid system. The nucleus remains in the zoospore cyst, which develops into a new sporangium and the anucleate rhizoids. Since the zoospore cyst retains the nucleus and develops into a sporangium, the sporangial development type is referred to as endogenous, and since this type of development results in a single sporangium per thallus, it is said to be monocentric. In some of the monocentric AF, such as the *Piromyces* spp., thallus development is of the exogenous type, which involves a two-sided germination of the zoospore cyst. During this process, a germ tube develops into a rhizoid system during the endogenous sporangial development, but once a substantial rhizoid has developed, a tubular outgrowth (the sporangiophore or sporangial stalk) emerges on the side opposite of the main rhizoid and the sporangium develops at the apex of the outgrowth. As the original nucleus escapes the zoospore cyst and develops elsewhere, the sporangial development is said to be exogenous (Barr et al., 1989). Both types are strictly determinate. In other members of the monocentric AF, such as the *Capellomyces*, both exogenous and endogenous sporangial development takes place (Hanafy et al., 2017, 2018, 2020; Joshi et al., 2018).

Polycentric AF display an exogenous thallus development, during which a one-sided germination of the zoospore cyst occurs. During the germination process, the content of the zoospore cyst migrates into germ tube which then develops into a nucleated branched rhizomycelium capable of developing multiple sporangia. Currently it appears that the remaining cyst, which has been emptied, has no further function. Development of thalli of polycentric fungi is said to be non-determinate.

In the case of the bulbous genera, nuclei are observed in the vegetative parts of the thallus (holdfast/branched sporangiophores), consistent with exogenous development. Thallus development in the bulbous genera is of a limited polycentric type, where the encysted zoospore forms a bulbous holdfast without rhizoids. Bulbous holdfasts give rise to single or multiple sporangia including branched sporangiophores. Growth in these fungi is not non-determinate like the thalli of polycentric fungi, but not as strictly determinate as in the case of the monocentric filamentous AF (Ozkose et al., 2001).

Our current understanding of the AF life cycle is based on what has been learned from rumen AF, but it is likely that AF associated with pseudoruminants pass through similar, if not even identical, life cycles stages. However, there are numerous aspects of AF biology that remain to be reassessed for hindgut herbivores. The cause of zoospore release in the hindgut is unclear, as it is not known if the known triggering compounds in the rumen (i.e., water-soluble haems and other porphyrins) can survive passage through the gastric stomach and small intestine. Whereas zoospores that are released within the rumen locate freshly ingested plant material chemotactically using soluble sugars (Orpin and Bountiff, 1978) and phenolic acids (Wubah and Kim, 1996), it is unclear to what extent these chemotactic signals are available to (and used by) AF in the cecum and colon of hindgut herbivores.

Regardless of herbivore type, the life cycle of AF has been proposed to contain a resting phase. While resting structures are still not fully understood, they provide a compelling explanation for why some of the currently known AF can be cultured from fecal material after prolonged periods of desiccation and oxygen exposure (Milne et al., 1989; Davies et al., 1993; McGranaghan et al., 1999; Griffith et al., 2009). To date resting cysts (Orpin, 1981), melanized resistant sporangia (Wubah et al., 1991) and multi-chambered spore-like structures (Brookman et al., 2000) have been described in different AF taxa. Although it may well be the case that there is no resting structure common to all AF, with taxon-specific structures instead, resting structures are thought to play an important role in the inter-animal transfer of AF. For example, it has been suggested that the survival of AF in saliva is likely to be an important transfer mechanism in ruminants and pseudoruminants (Lowe et al., 1987b). In hindgut fermenters, feces may play a more important role as a transfer mechanism between animals than saliva, particularly as certain hindgut fermenters, like foals, exhibit coprophagic behavior (Marinier and Alexander, 1995).

As zoospores and fungal thalli represent different parts of the same AF life cycle, consistent and accurate enumeration of AF is challenging. Approaches used to count exclusively free zoospores (France et al., 1990), fungal colonies on agar strips (Ushida et al., 1989), or both morphologies in culture supernatants (Theodorou et al., 1990; Obispo and Dehority, 1992; Griffith et al., 2009), and chitin measurements (Gay, 1991; Rezaeian et al., 2004b) have, in recent years, been widely replaced by molecular quantification via real-time PCR. This method overcomes the contrast within the life cycle between low zoospore numbers yet high AF vegetative biomass, as well as the paucity or absence of zoosporogenesis observed in some polycentric axenic cultures (Ho and Bauchop, 1991). On the other hand, the real-time PCR approach to quantify fungal biomass possesses its own challenges, such as the varying amount of fungal biomass produced by monocentric, polycentric and bulbous genera relative to DNA content. This makes translating quantitative estimates derived from real-time PCR into fungal biomass very challenging (Denman and McSweeney, 2006; Edwards et al., 2008). Estimating AF abundance by quantifying the number of ITS1 spacer regions [(Mura et al., 2019) or rRNA genes, i.e., 5.8S rRNA gene (Edwards et al., 2008) or LSU (Nagler et al., 2018)] seems to be taxon independent. However, it still remains to be determined if all AF have the same copy number of the *rrn* operon.

## Influence of Host on Anaerobic Fungi Community

Although diet has a significant effect on the structure of the AF community, host animal phylogeny has been shown to be a more important factor (Liggenstoffer et al., 2010; Kittelmann et al., 2012). Kittelmann et al. (2012) provided strong evidence that both diet and ruminant species, as well as interactions between those two parameters, affect the diversity of the AF community by subjecting cattle, sheep and deer to three different diets (summer pasture, winter pasture, and silage). Recent studies revealed that the genetic background of the host animal can influence the activity of the entire rumen microbiota, including the community of rumen AF (Roehe et al., 2016). In addition to the genetic background, several breed-associated phenotypes, such as eating frequency, dry matter intake, and rumen size potentially contribute to the variations in rumen microbiota that is observed among various breeds (Wallace et al., 2019; Zhang et al., 2020).

Anaerobic fungi were reported to be present in domesticated and non-domesticated equine species, with the AF community composition in horses and ponies being more similar to zebras than donkeys (Edwards et al., 2020b). In a separate study, AF diversity in donkeys was shown to be higher when compared to that of ponies and pony × donkey hybrids (Edwards et al., 2020a). Several studies have revealed that the genus *Khoyollomyces* (formerly known as AL1) is almost exclusively found in equines (Liggenstoffer et al., 2010; Mura et al., 2019; Edwards et al., 2020b; Hanafy et al., 2020). This may be due to the growth or metabolic characteristics of *Khoyollomyces*, making it more adapted to growth in the equine hindgut. Metabolic differences have previously been reported for equine and rumen strains of *Piromyces*, with equine strains possessing faster growth and higher fiber degradation capacity compared to rumen isolates (Julliand et al., 1998). This is perhaps not surprising considering the fundamental differences between ruminants (where freshly ingested feed directly enters the rumen), and hindgut herbivores (where feed first passes through the stomach and small intestine before reaching the hindgut) in terms of the main gut site where fiber degradation primarily occurs. There is also evidence indicating that the genus *Oontomyces* is specific to camelids (Dagar et al., 2015). Further research is needed to understand key factors that limit, or conversely broaden, the host distribution of certain AF taxa.

## Role of Anaerobic Fungi in Methanogenesis

Ruminants are a significant source of anthropogenic methane (CH_4_) producing ∼90 Tg of CH_4_ annually (Reay et al., 2018), with exact values differing based on the methodology employed to quantify emissions (Capper and Bauman, 2013; Lyu et al., 2018). Although methanogenesis occurs in the rumen, there appears to be no direct benefit of the microbial generated CH_4_ (which is released into the environment) to the animal itself. The ruminant provides the anaerobic environment that is necessary for the archaea, first described as archaebacteria in the late 1970s by Woese and Fox (1977), that are capable of CH_4_ production. Due to the complex nature of the rumen microbiome and the interactions between the individual community members and their biochemistry, the role of AF in methanogenesis can only be understood in light of knowledge about archaea and processes facilitated by them. There are three different biochemical routes by which archaea can produce CH_4_: (1) acetoclastic methanogenesis, (2) hydrogenotrophic methanogenesis, and (3) methylotrophic methanogenesis (Borrel et al., 2013; Offre et al., 2013).

Although most gut methanogens are thought to be hydrogenotrophic (Ferry, 2010; Borrel et al., 2013; Lyu et al., 2018), the majority of the global microbial CH_4_ is produced via acetoclastic methanogenesis (Ferry, 2010; Lyu et al., 2018). In the rumen ecosystem, both acetate and hydrogen are produced (Baldwin and Allison, 1983) and are available for methanogenesis. However, since their turnover rates in the rumen are high, the contribution of CH_4_ produced via the acetoclastic pathway accounts only for a small fraction of the overall CH_4_ produced in the rumen (Baldwin and Allison, 1983; Janssen, 2010). During hydrogenotrophic CH_4_ synthesis H_2_ and CO_2_ are combined to yield CH_4_ (Baldwin and Allison, 1983), with contributions from the newly described methylotrophic methanogens (Poulsen et al., 2013).

Under normal circumstances, H_2_ seldom reaches high concentrations in the rumen and the dissolved H_2_ concentration is usually about 0.1–50 μM, which is 0.014–6.8% of its maximum solubility at 39°C and 1 atm (Hegarty and Gerdes, 1999; Janssen, 2010). The scarcity and poor water solubility of H_2_ limits the access of methanogens to molecular hydrogen, necessitating the development of close physical contact and intimate syntrophic partnership between H_2_ producers and H_2_ metabolizers. Extreme forms of these interspecies H_2_-transfer are the methanogenic endo- and ecto-symbionts of rumen protozoa (Embley and Finlay, 1994). The close association seen between rumen ciliates and methanogens (Vogels et al., 1980) seems a more general feature of anaerobic ciliates, aimed at boosting their metabolic rate (Rotterová et al., 2020). Although AF are not known to have methanogenic endo- or ecto-symbionts, they do contain modified mitochondria known as hydrogenosomes (van der Giezen, 2009), and there is *in vitro* based evidence of cross-feeding (syntrophy) between hydrogenic AF and methanogenic archaea in the herbivore gut ecosystem (Yarlett et al., 1986).

Differential centrifugation of cellular fractions revealed that fungal hydrogenosomes convert malate or pyruvate under anaerobic conditions into H_2_, CO_2_, and acetate with the concomitant production of ATP (Yarlett et al., 1986; Marvin-Sikkema et al., 1993). This is similar to the process found in trichomonads, anaerobic urogenital parasites, where hydrogenosomes were first discovered (Müller, 1993). The H_2_ produced is the result of the action of the oxygen-sensitive enzyme hydrogenase (Davidson et al., 2002), which is the terminal electron acceptor for the metabolism coming from pyruvate. The excess H_2_ produced by AF can be used by methanogens to regenerate oxidized nucleotides (e.g., NAD, NADP) (Yarlett et al., 1986). Indeed, methanogenic archaea have even been found attached to the surface of fungal rhizoids and sporangia (Bauchop and Mountfort, 1981; Jin et al., 2011), which is likely to improve interspecies hydrogen transfer. Within the rumen microbiome, symbiotic ecto- and endosymbiotic partnership have been reported for rumen protozoa and methanogens (Finlay et al., 1994; Irbis and Ushida, 2004; Valle et al., 2015). Since other fungi have been shown to accommodate prokaryotic endosymbionts (Partida-Martinez et al., 2007), there is the possibility that methanogenic archaea can also exist intracellularly within AF, although such an intimate symbiotic relationship has not been reported until today.

There have been numerous co-culture studies, particularly with hydrogenotrophic *Methanobrevibacter* isolates and representatives of the genera *Piromyces*, *Neocallimastix*, *Orpinomyces*, *Caecomyces*, and *Anaeromyces* (Edwards et al., 2017). Methane, CO_2_, formate and acetate are the main products when AF are grown in the presence of methanogens, whereas H_2_, lactate, succinate and ethanol production is drastically decreased compared to the corresponding pure anaerobic fungal cultures (Bauchop and Mountfort, 1981; Marvin-Sikkema et al., 1990). This difference between co- and pure cultures is due to the inter-species hydrogen transfer in the methanogenic co-cultures influencing the efficiency of anaerobic fungal fermentation. This shifts AF product formation away from more oxidized end-products (i.e., lactate and ethanol) and toward the production of more reduced products (i.e., formate and acetate). Recently the life cycle stage has also been shown to shift metabolite production of AF grown in methanogenic co-culture (Li et al., 2019).

In exchange for the excess H_2_ produced by AF, methanogens have a beneficial effect on AF growth and activity. This is evidenced in terms of increased cellulolytic enzyme activity and dry matter disappearance in methanogenic co-cultures compared to anaerobic fungal cultures alone (Bauchop and Mountfort, 1981; Jin et al., 2011). The ability of AF and methanogen co-cultures to rapidly convert lignocellulose containing plant material into CH_4_ also has good potential for biotechnological applications (Cheng et al., 2009; Jin et al., 2011; Peng et al., 2016), particularly in terms of biogas production from lignocellulosic waste streams. Conversely, in ruminants CH_4_ production is viewed as an unfavorable outcome of the fermentation. In ruminants, eructation of H_2_ in the form of large amounts of CH_4_ represents a loss of energy for the animal in addition to being a significant source of anthropogenic CH_4_.

Several rodent hindgut fermenters and non-ruminant foregut fermenters also emit CH_4_ of a magnitude as high as ruminants, but in contrast equids, macropods and rabbits produce much less (Clauss et al., 2020). There has been a move in recent years to understand why some domesticated ruminants produce lower CH_4_ compared to others (Shi et al., 2014), and a subsequent push to utilize feed-based approaches to decrease ruminal methanogenesis (Jeyanathan et al., 2014). However, it remains to be determined to what extent the growth and activity of rumen AF is affected by reducing the number and/or activity of methanogenic archaea using these approaches. Being able to understand the interdependencies of these populations will be important for informing a holistic understanding of the microbiome as it pertains to ruminant function. Current knowledge regarding interactions of AF with bacteria and protozoa is more limited, and has been reviewed elsewhere (Gordon and Phillips, 1993; Edwards et al., 2017).

## Role of Anaerobic Fungi in Plant Cell-Wall Degradation

*In vitro* studies suggest that the contribution of AF to the ruminal degradation of plant material could be more significant than the contribution of cellulolytic rumen bacteria (Joblin et al., 1989; Lee et al., 2000b). This is likely due to the broad range of enzymes that are produced by the AF combined with their physical ability to break open fibrous materials through their penetrating hyphal tips. These tips have high concentrations of fibrolytic enzymes, whose enzymatic activity subsequently also increases nutrient access for other cellulolytic microbes (Ho et al., 1988; Ljungdahl, 2008; Haitjema et al., 2014; Dagar et al., 2015; Solomon et al., 2016). Whilst rumen zoospore numbers are low compared to counts of bacteria and archaea, AF have been shown to represent up to 20% of the rumen microbial biomass (Rezaeian et al., 2004a) and 10–16% of rRNA transcript abundance (Elekwachi et al., 2017). The observation that some AF species are capable of releasing up to 95% of the fermentable sugars from untreated plant leaves during a 4-day incubation period (Sijtsma and Tan, 1996) further highlights their critical role during the rumen digestion of fibrous plant biomass. These findings have led to the general belief that AF have been essential in the successful evolution of mammalian herbivores (Wang et al., 2019).

Understanding how AF and their carbohydrate-active enzymes (CAZymes) contribute to the degradation of indigested biomass in the herbivore gut is rather limited compared to the knowledge of the role of bacteria. This is partially due to the more extensive genomic resources that exist for rumen bacteria compared to AF (Hess et al., 2011; Seshadri et al., 2018; Stewart et al., 2019). AF genome information that is currently publicly available is summarized in Table 2, and includes the AF strain host isolation source and number of CAZymes identified.

**TABLE 2:** Publicly available genomes of anaerobic fungi.

**Organism**	**Strain**	**Host**	**Genome size^*a*^ [base pairs]**	**Gene count^*a*^**	**CAZyme count^*a*^**	**References**
*Anaeromyces robustus*	S4	Sheep	71,685,009	12,832	1,766	Haitjema et al. (2017)
*Caecomyces churrovis*	–	Sheep	165,495,782	15,009	ND^*b*^	Henske et al. (2017)
*Neocallimastix californiae*	G1	Goat	193,495,782	20,219	2,743	Haitjema et al. (2017)
*Pecoramyces ruminantium*	C1A	Cow	100,954,185	18,936	2,029	Youssef et al. (2013)
(formerly *Orpinomyces* sp.)						
*Piromyces finnis*	Pirfi3	Horse	56,455,805	10,992	1,463	Haitjema et al. (2017)
*Piromyces* sp.	E2	Elephant	71,019,055	14,648	3,819	Haitjema et al. (2017)

*^*a*^https://mycocosm.jgi.doe.gov (Grigoriev et al., 2013). ^*b*^Not determined.*

One striking feature of AF besides their large repertoire and diversity of CAZymes (Supplementary Table S1), is the ability of their CAZymes to form cellulosomes. Cellulosomes, first identified in anaerobic bacteria, are extracellular multi-enzyme complexes that tether together an assortment of cellulases and related accessory enzymes (Lamed et al., 1983). The assembly of these AF multi-protein complexes, in some cases with individual building blocks from different species, is facilitated by fungal dockerins, which can directly bind to plant cell wall components without the need for a scaffoldin (Fanutti et al., 1995). This is in stark contrast to bacterial cellulosomes, which are highly species-specific (Bayer et al., 2004).

Cellulosomes have been linked to the improved fitness and biomass-degradation phenotype of both anaerobic bacteria and AF (Bayer et al., 2004; Henske et al., 2018) by enabling the synergistic activity of the individual biomass-degrading enzymes. Cellulosomes have been shown to increase cellulolytic activity over free enzymes by up to 12-fold (Krauss et al., 2012). Synthetic cellulosomes, inspired by bacterial cellulosomes, have shown promise for industrial applications due to outperforming free cellulolytic enzymes when produced in recombinant systems (Gilmore et al., 2020). Although evidence of AF cellulosomes emerged about 20 years ago (Wilson and Wood, 1992), it is only recent work that has provided strong support for the hypothesis that these multi-enzyme machineries might hold the secret to the superior biomass-degrading capability of AF. However, cellulosomes from AF still remain poorly understood (Haitjema et al., 2017).

Horizontal gene transfer (HGT) between the different populations of rumen microbes appears to have played a significant role in the evolution of the Neocallimastigomycota (Murphy et al., 2019). HGT between rumen bacteria and rumen AF was proposed by Garcia-Vallvé et al. (2000) as a major mechanism that allowed rumen AF to acquire many of the CAZymes that have now been identified in their genomes (Supplementary Table S1). Since then, numerous other authors have supported the hypothesis that the acquisition of CAZymes from bacterial donors enabled the rumen AF, as well as rumen protozoa (Ricard et al., 2006), to successfully compete for plant carbohydrates in the herbivore gut (Duarte and Huynen, 2019; Wang et al., 2019). Despite the numerous CAZyme families that AF acquired via HGT, the cellulosome of AF appears to have unique attributes that distinguish it from those that are found in gut bacteria.

Cellulosomes, regardless of their origin, are typically composed of several enzymes (i.e., cellulases and hemicellulases) that contain an active site, one or several carbohydrate binding modules and a dockerin that facilitates the “docking” of the enzymatic multi-modular complex to one of the multiple cohesins that are displayed by a scaffoldin. In anaerobic bacteria, cellulosomes are anchored to the bacterial cell wall via dockerin-scaffoldin modules (Fontes and Gilbert, 2010). In contrast, AF cellulosomes are not necessarily anchored to the fungal cell wall, but can be also found as free multi-enzyme complexes that are released into the extracellular matrix (Haitjema et al., 2014). These free cellulosomes enable an increased concentration of enzymes that possess catalytic and carbohydrate binding sites without the need to have them linked to and displayed on the AF cell surface. More importantly, it has been suggested that these free cellulosomes are non-species specific and theoretically enable the synthesis of cellulosomes using components of different donors (Nagy et al., 2007).

Another aspect that distinguishes AF cellulosomes from their bacterial counterparts is the presence of modules that have sequence signatures which are conserved within members of glycoside hydrolase (GH) families GH3, GH6, and GH45 (Haitjema et al., 2017). The ability to expand the substrate repertoire of AF cellulosomes by incorporating these GH signatures might provide additional enzymatic capabilities not conveyed by bacterial cellulosomes, such as the conversion of cellulose to monosaccharides by the β-glucosidase activity of GH3.

**FIGURE 2:**
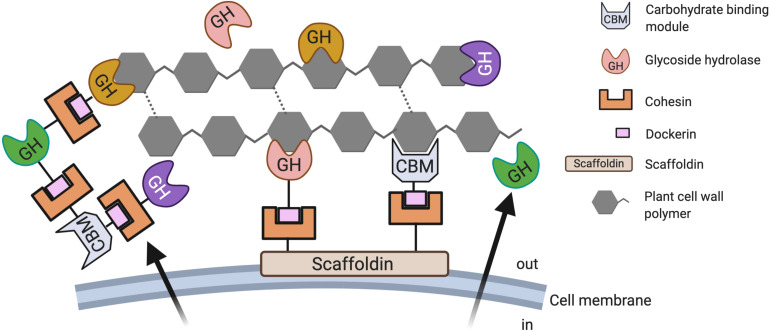
Carbohydrate-active enzymes employed by anaerobic fungi during biomass conversion. Anaerobic fungi (AF) deploy various strategies for the degradation of plant biomass. It has been suggested that their ability to produce secreted free CAZymes, cell-bound multi-enzyme complexes (cellulosomes), as well as free cellulosomes might provide the AF with the competitive advantage over the CAZyme repertoire produced by anaerobic bacteria (Henske et al., 2017).

Besides cellulosomes, free CAZymes are also produced AF, which ultimately leaves the AF with several hydrolytic mechanisms to attack plant cell wall polymers from multiple directions (Haitjema et al., 2017) (Figure 2). The ability to produce secreted free CAZymes, cell-bound cellulosomes, as well as free cellulosomes might provide the AF with the competitive advantage over the CAZyme repertoire produced by the cellulolytic anaerobic bacteria also resident in the herbivore gut (Henske et al., 2017). It has also been proposed that the superior efficiency in biomass degradation of AF might be caused to a great extent by the ability to simultaneously employ a diverse set of cellulosome-bound as well as free (not cellulosome associated) CAZymes that also display a significant functional overlap (Couger et al., 2015).

This hypothesis, as well as the ability of AF to contribute a complementary set of CAZymes to the ones provided by bacteria was recently supported by genome-centric metaproteome and metatranscriptome approaches (Hagen et al., 2020). Shotgun metaproteome and metatranscriptome data were mapped back on previously assembled rumen genomes from prokaryotes and cultured AF, as well as on Metagenome Assembled Genomes to determine the origin of the different CAZymes detected in the microbiome that colonized recalcitrant plant material during rumen-incubation (Hagen et al., 2020). Results from this study revealed that the bacterial population contributed CAZymes mostly associated with the degradation of more readily degradable carbohydrates such as hemicellulose, whereas fungi provided CAZymes (e.g., enzymes belonging to the GH family GH5, GH6, GH8, and GH48) that targeted the more recalcitrant plant cell wall components. These data also provided clear evidence for the involvement of AF cellulosomes in the biomass degradation process. It can be assumed that a more detailed understanding of AF and their enzymatic repertoire, in the context of a highly diverse and synergistically working microbial ecosystem, will emerge as more of these complex and large-scale meta-omics data sets are generated and analyzed.

## Anaerobic Fungi as Feed Additives to Promote Animal Health and Performance

The capability of AF to colonize and degrade otherwise recalcitrant plant structures via a set of highly efficient enzymes has led to an increased interest in the application of AF, and their enzymes, to boost the digestibility of low-quality feed and to increase the overall feed efficiency of herbivorous animals. Enabling the utilization of low-quality feed will be essential to enable the production of high-quality animal proteins, especially during a time when terrestrial areas for the production of high-quality feed will become scarce due to a changing climate and the rapid urbanization of areas that are currently being used to grow feed crops.

Several *in vivo* studies indicated that continuous dosing of ruminants with live culture of AF results in the changes of numerous parameters indicative of enhanced feed digestibility and feed efficiency, with benefits of AF dosing appearing to be more pronounced in young ruminants (Sehgal et al., 2008). Measurements performed to determine the animal’s ability to digest feedstuff in response to AF dosing, include dry matter (DM), neutral detergent fiber (NDF) and acid detergent fiber (ADF) digestibility, animal growth, milk yield and VFA concentrations (all parameters increased when AF were added) (Dey et al., 2004; Paul et al., 2004, 2011; Tripathi et al., 2007; Saxena et al., 2010).

Probiotic capabilities of live AF cultures for ruminants were indicated in the 1990s when living AF cultures were added to the rumen of cattle and sheep from which fungi had been previously removed. Forage intake by such fungus-free early weaned calves was 35% higher in those that had been dosed with *Neocallimastix* sp. R1 (Theodorou et al., 1990), and dosing of fungus-free sheep with *Neocallimastix* sp. SLl resulted in a 40% increase in intake of a straw based diet (Gordon and Phillips, 1993).

Despite the work that has been conducted to investigate how dosing the rumen ecosystem with AF cultures affected the abundance of the native fungal, bacterial and even the ciliate rumen populations (Lee et al., 2000a; Paul et al., 2004, 2011), there has been no evidence that suggests that dosing with AF, and therefore the increase of AF concentration in the rumen, resulted in a decrease of bacteria or ciliate protozoa nor in a drop of feed digestibility. In contrast to this, a positive correlation between fungal and bacterial concentrations, most likely due to the fact that the hyphae of AF physically open the plant tissue thereby increasing surface area available for colonization and nutrient access for other fibrolytic rumen microbes, has been reported (Bauchop, 1979; Akin and Borneman, 1990). This increased accessibility would also explain why counts of the holotrichs, the starch degrading protozoal population, increased in response to dosing with AF, while the overall count of the ciliate rumen protozoa remained stable (Paul et al., 2004).

Besides the lack of evidence that an increase in AF by dosing reduces the abundance of other rumen populations, it appears to be noteworthy that none of the studies centering on AF dosing has looked at potential changes of fiber-colonization by AF after the dosing event. Considering that dosing with AF cultures improved fiber digestion, VFA production and animal performance related parameters, suggesting that the altered microbiome is more efficient in the digestion of fibrous feeds than the original community, a closer investigation of the fiber colonization and deconstruction process post-dosing seems warranted.

Whereas ruminal fiber digestion improved in response to live AF, no shift in fermentation pattern was observed when AF-derived enzymes were added to the diet (Lee et al., 2000a). This highlights the importance of using viable cultures of AF as ruminant feed additives (Paul et al., 2011). In contrast to this, spent media containing AF enzymes seems to be effective in improving monogastric livestock production (Theodorou et al., 1996). Cell walls of cereals, major components of swine and poultry feed, contain difficult to digest non-starch polysaccharides, such as β-glucans in barley and wheat and arabinoxylans in rye and oats (Sánchez-Rodríguez et al., 2012). These polymers can have anti-nutritional effects due to their low digestibility and tendency to form high-molecular-weight aggregates that reduce passage rate, decrease diffusion of digestive enzymes, promote endogenous losses, and stimulate unwanted bacterial proliferation (Bedford and Schulze, 1998). Previous studies suggest that recombinant GHs from AF expressed in *Lactobacillus reuteri* maintain their fiber-degrading capability and their resistance to bile salt and acids, while *L. reuteri* itself still retained its high adhesion efficiency to mucin and mucus (Liu et al., 2005a,b, 2007; Cheng et al., 2014), which would explain the decomposition-promoting effect of these recombinant proteins in the monogastric animal. Despite these promising characteristics for recombinant AF-derived GHs, the ability to efficiently generate significant amounts of cheap, stable, and active recombinant enzymes will be essential for low-cost production and large-scale application of these enzymes as a feed additive for monogastrics.

Tannins present in many feeds and forages are inhibitory to rumen microbes, and are an anti-nutritional issue for ruminants. Likewise, upon anaerobic degradation of phenolic compounds present in fibrous feeds, different phenolic monomers (i.e., ferulic acid, *p*-coumaric acid, vanillic acid, vanillin, catechol etc.) are released into the rumen that are inhibitory to microbiota. Therefore, attempts were made to identify rumen microbes that had tannin or phenolic monomer tolerating or degrading capability, so that they could be utilized as direct fed microbials to mitigate adverse effects of these anti-nutritional factors.

The anaerobic rumen fungus *Piromyces* sp. FNG5, isolated from a wild herbivore, was found to be tolerant to phenolic monomers and its pure culture degraded p-coumaric acid (38.5–65.1%), 65.2–74.1% ferulic acid (65.2–74.1%) and vanillic acid (34.1–66.8%) after 14 days of incubation (Paul et al., 2003). McSweeney et al. (2001) reported that sheep fed with condensed tannins from *Calliandra calothyrsus* had reduced ruminal AF concentration, but the inhibitory effect was less prominent compared to rumen bacteria. Paul et al. (2006) reported that addition of *Piromyces* sp. FNG5 significantly increased *in vitro* degradation (12%) of condensed tannins and this AF isolate could tolerate tannic acid concentrations up to 20 g/L. This amount is higher than the theoretic tannic acid level expected in the rumen of animals fed a diet composed exclusively of high tannin content plants. Conversely, Kok et al. (2013) found that *L. leucocephala* hybrid-Bahru (containing condensed tannins) when fed to goats, significantly decreased ruminal AF concentration. Saminathan et al. (2019) reported that high MW fractions of condensed tannins had inhibitory effect on ruminal AF, but relative abundance of *Piromyces* 4 was increased indicating that this group of uncultured AF is likely to be tannin resistant. The mechanisms by which some AF species can overcome the growth inhibitory effects of condensed tannins or phenolic monomers is unknown. Whether AF produce tannase, or not, remains to be established; but many isolates of AF, especially those from wild ruminants adapted to tannin rich and fibrous diets, were shown to produce a variety of esterases capable of degrading phenolic compounds (Paul et al., 2003). It is possible that these esterases are directly linked the ability of these AF to overcome growth inhibition caused by phenolic compounds.

Although these studies highlight the potential of using AF as probiotics to enhance digestibility of highly fibrous or tanniferous feed, boosting ruminant livestock production, economic aspects such as the need to repeatedly administer oral-dosages of AF to maintain the desired response have to be considered when discussing AF as probiotics on a commercial scale (Ribeiro et al., 2016).

As well as positively impacting forage intake and feed digestibility, AF have the potential to contribute to the protein supply of the host animal. This is both indirectly through the production of proteolytic enzymes in the rumen and directly as a source of microbial protein. Unlike the cellulolytic rumen bacteria, many isolates of AF are protease positive and capable of penetrating the proteinaceous layer of feed particles (Wallace and Joblin, 1985; Asao et al., 1993; Michel et al., 1993; Yanke et al., 1993). *In vitro* studies with defined populations of both proteolytic and non-proteolytic rumen bacteria and a proteolytic *Neocallimastix frontalis* strain have further indicated that *N. frontalis* was able to contribute to rumen protein degradation, particularly when protein was associated with feed particles (Wallace and Munro, 1986).

Besides producing proteases, AF directly contribute to protein supply of the host in terms of being part of the microbial biomass that passes down to the intestines from rumen, for subsequent digestion and absorption. Gulati et al. (1989) showed that AF cells were composed of proteins with a well-balanced combination of amino acids that were highly available to the ruminant host. A high proportion of the protein components of three monocentric AF (i.e., *Neocallimastix* sp. LMI, *Piromyces* sp. SMI and *Caecomyces* sp. NMI) was digested and absorbed in the intestine of sheep, with digestibility factors of 0.91–0.98 (Gulati et al., 1988, 1989). These high *in vivo* digestibility values for AF protein compared favorably with a value of 0.77 for mixed rumen bacteria protein measured in a similar manner (Gulati et al., 1990). Although the amount of nutritional nitrogen derived directly from anaerobic rumen fungi might only amount to a small portion of the total nitrogen that is absorbed by the animal, its importance lies in its high quality and immediate availability.

## Dietary Manipulation of Commensal Anaerobic Fungi

Rather than directly adding more AF to the ruminant animal, there have been numerous attempts to improve the concentration of AF in the rumen by providing animals with feed that increases their overall concentration. It was previously believed that increasing dietary recalcitrant fiber may increase fungal population in rumen, as some of the plant fiber breakdown products were shown to have a positive effect on zoosporogenesis and chemotactic effects on fungal zoospores. Few AF were seen in the rumen of animals that were fed lush pasture (i.e., legume or grass when green and leafy), and the number of AF increased when animals were fed the same pasture after it had matured and was more recalcitrant (Bauchop, 1989; Kostyukovsky et al., 1991). However, results later showed the opposite effect, with an increased AF count in ruminants fed a low-lignin diet reported compared to animals provided with more recalcitrant feed (Gordon and Phillips, 1998). To make matters even more complex, other studies suggested that there was no direct effect of the hay type (with different levels of lignin content) on AF populations (Sekine et al., 1995).

It has been suggested that an appropriate amount of starch or concentrate in diet may support ruminal AF growth and stimulate zoosporogenesis (Matsui et al., 1997), but *in vivo* study findings remain inconclusive (Ishaq et al., 2017). A possible explanation for these different responses to starch rich feed is that only some of the AF, namely species of the genera *Neocallimastix*, *Piromyces* and *Orpinomyces*, have been shown to produce amylases and, therefore, have the ability to ferment starch grains (Phillips and Gordon, 1988; McAllister et al., 1993; Yanke et al., 1993). More work in this area needs to be conducted *in vivo* before final conclusions can be made. Furthermore, feeding starch or concentrates tends to increase ruminal ciliate protozoal concentrations, and protozoa are known to predate on AF zoospores (Morgavi et al., 1994).

Beneficial effects of sulfur supplementation, specifically for low sulfur diets, on the number of AF in sheep and their relative contribution to fiber degradation was reported in the early 1980s (Akin et al., 1983). This beneficial effect was further confirmed in subsequent studies using alkali treated wheat straw (Gordon et al., 1983; Gulati et al., 1985; Weston et al., 1988) and other poor quality feeds (Morrison et al., 1990). Promkot and Wanapat (2009) suggested beneficial manipulation of AF concentrations was possible through provision of an appropriate dietary supplement containing sulfur. For a supplement of this type to be effective, it should ideally contain a single organic sulfur compound which is readily utilized by the rumen AF but not by other components of the rumen microbiota (i.e., bacteria, archaea, and protozoa).

Two organic sulfur nutrients, mercapto-1-propionic acid (MPA) and 3-mercapto-1-propanesulfonic acid, were tested in cattle trials and compared to an inorganic sulfur supplement. It was reported that the organic sulfur sources improved nitrogen utilization and microbial protein production, but surprisingly this was concluded to be due to a general improvement in the efficiency of microbial fermentation of lignocellulose and not from specific stimulation of ruminal AF (McSweeney and Denman, 2007). Conversely, in a patent (Gordon and Phillips, 2002) it was reported that administering an effective amount of a degradation resistant sulfur source (MPA or its functional equivalent) promoted the growth of AF in the rumen of animals fed low sulfur content diets. Within this patent, it was also demonstrated that ruminal MPA infusion increased AF zoospores concentrations, and had a strong, positive, response on the digestive performance of sheep. However, additional scientific literature in this field is scarce and a sulfur supplement specific for promoting anaerobic rumen fungi remains to be identified.

Influence of other dietary supplements on the rumen AF community has been less studied, but some interesting findings have been reported. Thiamine supplementation, used to attenuate rumen metabolic disorder caused by high concentrate diet through buffering the rumen pH, increased significantly the proportion of ruminal AF in dairy cows (Xue et al., 2018). Plant oils, which are attractive feed additives used to mitigate CH_4_ emissions, seem to have a negative effect on AF. The addition of soya oil significantly reduced ruminal AF diversity in steers (Boots et al., 2013). In dairy cows, sunflower oil addition decreased the concentration and diversity of AF. However, responses at the genus level were dependent on concentrate/forage ratios (Tapio et al., 2017). The addition of rapeseed oil led to a considerable decrease in the ruminal AF population, but the mechanism was not further investigated (Fonty and Grenet, 1994). Previously, Elliott et al. (1987) found that feeding a supplement of sunflower meal to sheep consuming a barley straw diet resulted in decrease of ruminal AF concentration to below detectable levels. In another study, the feeding of calcium salts of medium chain fatty acids (C6–Cl2) to sheep resulted in reduced numbers of AF zoospores in the rumen, whereas the salts of long chain fatty acids (C ≥ 14) had no effect on AF (Ushida et al., 1992). This indicates that the inhibitory effects of the long chain fatty acids common in oilseed meals can be alleviated, at least partly, by chemical pretreatment.

Anaerobic fungi are also sensitive to a shortage of nitrogen. A low protein diet decreased rumen AF concentration in dairy cows compared to a high protein diet (Belanche et al., 2012). However, the AF community composition was modified by the level of dietary protein only when cows consumed the starch-rich diet, but not the fiber-rich diet (Belanche et al., 2012). This highlights the potential for further complexities when trying to determine the effects of individual dietary components on ruminal AF.

## Future Perspectives and Opportunities for Anaerobic Fungi Based Applications in Animal Production and Health

With consumer demand for affordable high-quality animal products increasing and with a decline in natural resources, such as farmable land area, it will be essential to create new and refine existing strategies to improve the utilization of low-quality forages for animal feed. Such strategies will rely heavily on approaches that render the recalcitrant fraction of the plant material more accessible to the fermenting microorganisms that are indigenous to the herbivore gut. AF with their ability to break open recalcitrant plant structures will play a significant role in these new approaches.

Whilst AF are clearly beneficial for the ruminant host, the underlying mechanisms to boost indigenous AF populations through standard feed components such as fiber, starch, nitrogen and lipid are inherently complex and are still not well understood. As such, there still remains great interest in developing a reliable and reproducible feed-based strategy to increase AF in order to improve animal performance and health. However, whilst the benefits of AF for ruminants has been well established, their value for hindgut herbivores remains to be confirmed. The observed increase in feed intake with AF supplemented ruminants is thought to be due to the AF causing more rapid clearance of digesta from the rumen, due to their physical and enzymatic disruption of fibrous plant particles (Gordon and Phillips, 1998). If this is also the case in equines, development of an equine AF probiotic may enable replacement of some of the energy dense concentrates used in horse feeds with more bulky fibrous feeds. This will contribute to reducing the risk of colic and dysbiosis of hindgut microbiota, which is commonly observed in working and/or performance equines fed high grain/concentrate diets in order to meet their higher energy requirements (Shirazi-Beechey, 2008; Durham, 2013; Julliand and Grimm, 2017).

Most of the studies focused on AF-based strategies for improving animal production and health have relied on the repeated oral-dosing of AF. This approach can only become economically feasible if the cost of industrial scale production of an AF probiotic is significantly less than the economic return gained by livestock producers. Considering the significant benefits that have been reported for live AF supplementation to date for ruminants, the probiotic use of AF is likely to have a significant return on investment for ruminant livestock producers. One possibility to produce an AF probiotic could be the use of encapsulated cultures. However, methods for large-scale production of encapsulated AF are currently not available, and would have to be developed before this approach could become commercially feasible. An alternative approach could be the use of AF resting structures, with their subsequent revival into active hydrolytic AF occurring within the host. Fundamental understanding of these structures is, however, currently too limited for this to be practically realized in the near future. Advances in understanding of the biology of the AF resting phase would not only facilitate the utilization of AF in livestock production, but also their application in other areas where lignocellulosic plant material is used to produce biofuels and platform chemicals. As such, characterization of the resting phase of AF should be a high priority research area for development.

The resulting increased efficiency of livestock production through the application of AF will undoubtedly have beneficial impacts in terms of the environmental footprint and sustainability of livestock production. The impact of AF based strategies on ruminant derived methane production remains to be determined, however, it is clear that increased efficiency of lignocellulosic plant material utilization will also decrease the need for arable land for animal feed production. Recent advances in molecular techniques enable a detailed understanding of role of the AF and how it affects the performance and health of its herbivorous host. Research based on state-of-the-art methods will allow the development of more advanced and holistic approaches to manipulate the composition and function of the gut microbiome and ultimately the health and performance of the host animal. Such progress will facilitate a more sustainable livestock industry to provide affordable high-quality animal products for a growing global population.

## Conclusion

Although AF were first observed more than hundred years ago, it was not until Colin Orpin’s ground-breaking work in the mid 1970s that AF were correctly identified, overturning the paradigm of the time that all fungi required oxygen. Considering AF have only been effectively studied for ∼50 years, advances made during this time have been significant. Whereas initial work required the refinement of isolation and cultivation techniques and was mostly driven by morphological observations and phenotypic characterization, more recent insights have been enabled by novel molecular approaches. These molecular techniques have already greatly advanced understanding of the complexity and diversity of the AF, however, our full understanding is still far from complete. With the ability to sequence long genomic regions such as the entire *rrn* operon, it is inevitable that a more accurate and complete understanding of the AF phylogeny will soon emerge. Understanding the phylogenetic relationship of individual AF will be essential to increasing understanding of their evolutionary history, and factors that drive their niche specialization within and between different types of host. However, to practically develop AF for application in livestock production, as well as other industries, functional systems microbiology approaches will be key. Technologies such as (meta)genomics, (meta)transcriptomics and proteomics will enable the pinpointing of specific genes, proteins and reactions that are employed by AF in response to extrinsic conditions (e.g., host genotype) and changes therein (e.g., host health status and dietary composition). Despite the significant progress made to date, the ecological role of AF and their quantitative contribution to host function and health still remains to be clarified in the full range of mammalian herbivores where they naturally reside. Furthermore, a key phase of the AF life cycle, the resting phase, is an area of very limited knowledge that urgently needs to be researched. Together, newly obtained knowledge in these areas will enable utilization of AF and their enzymes to transform the sustainability and environmental footprint of livestock agriculture, as well as revolutionizing biotechnological processes involving plant-based feedstocks. Whilst we increasingly understand more about the evolution, biology and ecology of AF, there still remains many key “why” questions to be answered: “Why” are AF sensitive to oxygen? “Why” do AF have the lowest GC-content among all known microorganisms? “Why” are AF the only fungi with polyflagellate zoospores, hydrogenosomes, and cellulosomes? Considering that the AF phylum is currently composed of just one family, are we only looking at the tip of an iceberg? Or have we missed something crucial when classifying these microorganisms? Whatever the answers are, we know for certain that many fundamental questions still remain to be answered before the true potential of this highly valuable and paradigm shifting phylum of microorganisms is fully understood and can be harnessed.

## Author Contributions

MH designed the manuscript, coordinated the co-author contributions, and wrote the plant cell-wall degradation section. KF wrote the habitat, life cycle, morphology and taxonomy section and contributed to other sections. SP and AP contributed to the feed additive section. MG contributed to the methanogenesis section. CS co-authored the plant cell wall degradation section. JE wrote the section on hindgut herbivores and contributed to other sections. All authors reviewed the manuscript, offered critical feedback, and approved the final version.

## Conflict of Interest

During the development of the manuscript, JE changed employment from Wageningen University & Research to the company Palital Feed Additives, Netherlands. The remaining authors declare that the research was conducted in the absence of any commercial or financial relationships that could be construed as a potential conflict of interest.

## Footnotes

1 https://www.anaerobicfungi.org
