# Supplementary material for: Anaerobic Fungi: Past, Present, and Future
Source: Front Microbiol. 2020 Oct 21;11:584893. doi: 10.3389/fmicb.2020.584893 (PMC7609409; doi:10.3389/fmicb.2020.584893)
Supplement: Supplementary file 1 [file Table_1.DOCX]

**Supplemental Table 1: Carbohydrate-Active Enzyme profiles of anaerobic fungi.**

|  |  | **Organism** | | | | |
| --- | --- | --- | --- | --- | --- | --- |
|  |  | *Anaeromyces robustus* | *Neocallimastix californiae* | *Pecoramyces ruminantium* | *Pecoramyces ruminantium* | *Piromyces finnis* |
| **Carbohydrate-Binding Module (CBM) Families** | CBM1 | 94 | 144 | 104 | 102 | 103 |
|  | CBM6 | 6 | 14 | 7 | 14 | 7 |
|  | CBM10 | 12 | 33 | 29 | 2 | 20 |
|  | CBM12 | 1 | 2 | 2 | 3 | 6 |
|  | CBM13 | 20 | 48 | 23 | 12 | 17 |
|  | CBM18 | 447 | 519 | 500 | 640 | 288 |
|  | CBM21 | 4 | 8 | 4 | 2 | 5 |
|  | CBM22 | 1 | 1 | 0 | 0 | 1 |
|  | CBM25 | 11 | 4 | 13 | 1 | 1 |
|  | CBM26 | 20 | 10 | 26 | 15 | 1 |
|  | CBM29 | 2 | 15 | 12 | 7 | 18 |
|  | CBM32 | 0 | 5 | 1 | 2 | 0 |
|  | CBM35 | 3 | 7 | 2 | 6 | 3 |
|  | CBM48 | 8 | 17 | 5 | 5 | 9 |
|  | CBM50 | 5 | 4 | 4 | 4 | 5 |
|  | CBM52 | 5 | 11 | 5 | 3 | 5 |
|  | CBM61 | 1 | 3 | 1 | 1 | 1 |
|  | CBM63 | 6 | 7 | 3 | 2 | 3 |
|  | CBM66 | 2 | 0 | 3 | 0 | 0 |
|  | **Carbohydrate-Binding Modules (CBMs)** | **648** | **852** | **744** | **821** | **493** |
| **Carbohydrate Esterase (CE) Families** | CE1 | 28 | 48 | 33 | 37 | 17 |
|  | CE2 | 11 | 8 | 6 | 10 | 1 |
|  | CE3 | 1 | 1 | 1 | 3 | 0 |
|  | CE4 | 46 | 88 | 45 | 41 | 42 |
|  | CE6 | 11 | 14 | 8 | 18 | 11 |
|  | CE8 | 5 | 14 | 9 | 8 | 5 |
|  | CE12 | 6 | 8 | 2 | 7 | 6 |
|  | CE15 | 2 | 6 |  | 3 | 2 |
|  | CE16 | 9 | 24 | 14 | 11 | 6 |
|  | **Carbohydrate Esterases (CEs)** | **119** | **211** | **118** | **138** | **90** |
| **Glycoside Hydrolase (GH) Families/Subfamilies** | GH1 | 7 | 16 | 10 | 17 | 10 |
|  | GH2 | 1 | 7 | 1 | 1 | 1 |
|  | GH3 | 15 | 53 | 18 | 26 | 15 |
|  | GH5 | 26 | 72 | 50 | 46 | 29 |
|  | GH5_1 | 4 | 6 | 6 | 6 | 3 |
|  | GH5_2 | 1 | 0 | 0 | 0 | 0 |
|  | GH5_4 | 12 | 34 | 22 | 22 | 19 |
|  | GH5_5 | 3 | 12 | 7 | 2 | 4 |
|  | GH5_7 | 6 | 19 | 8 | 12 | 3 |
|  | GH5_8 | 0 | 1 | 4 | 3 | 0 |
|  | GH5_54 | 0 | 0 | 1 | 0 | 0 |
|  | GH6 | 13 | 28 | 49 | 35 | 22 |
|  | GH8 | 2 | 2 | 1 | 2 | 1 |
|  | GH9 | 9 | 14 | 13 | 12 | 12 |
|  | GH10 | 15 | 60 | 32 | 29 | 21 |
|  | GH11 | 33 | 30 | 52 | 72 | 41 |
|  | GH13 | 12 | 13 | 13 | 10 | 6 |
|  | GH13_8 | 3 | 5 | 3 | 3 | 3 |
|  | GH13_14 | 1 | 2 | 0 | 1 | 1 |
|  | GH13_23 | 1 | 0 | 0 | 0 | 0 |
|  | GH13_25 | 1 | 1 | 1 | 1 | 1 |
|  | GH13_28 | 5 | 4 | 8 | 3 | 0 |
|  | GH16 | 12 | 21 | 6 | 6 | 8 |
|  | GH18 | 12 | 17 | 9 | 15 | 9 |
|  | GH20 | 1 | 2 | 1 | 1 | 1 |
|  | GH24 | 2 | 1 | 3 | 3 | 7 |
|  | GH25 | 2 | 2 | 4 | 3 | 4 |
|  | GH26 | 3 | 15 | 1 | 9 | 4 |
|  | GH28 | 0 | 1 | 0 | 1 | 0 |
|  | GH30 | 2 | 4 | 3 | 3 | 1 |
|  | GH30_3 | 0 | 1 | 0 | 0 | 0 |
|  | GH30_5 | 2 | 3 | 2 | 3 | 1 |
|  | GH31 | 7 | 10 | 19 | 5 | 2 |
|  | GH32 | 2 | 11 | 1 | 1 | 0 |
|  | GH36 | 1 | 1 | 1 | 1 | 1 |
|  | GH37 | 1 | 1 | 2 | 1 | 1 |
|  | GH38 | 2 | 1 | 2 | 2 | 1 |
|  | GH39 | 5 | 9 | 3 | 5 | 2 |
|  | GH43 | 18 | 47 | 32 | 31 | 14 |
|  | GH45 | 14 | 28 | 16 | 20 | 15 |
|  | GH47 | 5 | 6 | 1 | 3 | 2 |
|  | GH48 | 7 | 21 | 14 | 14 | 13 |
|  | GH53 | 1 | 3 | 0 | 2 | 1 |
|  | GH64 | 1 | 4 | 2 | 1 | 1 |
|  | GH67 | 0 | 0 | 0 | 1 | 0 |
|  | GH73 | 0 | 1 | 0 | 0 | 0 |
|  | GH74 | 1 | 4 | 2 | 4 | 3 |
|  | GH78 | 1 | 1 | 2 | 1 | 0 |
|  | GH95 | 2 | 2 | 0 | 1 | 1 |
|  | GH97 | 0 | 1 | 0 | 0 | 0 |
|  | GH105 | 0 | 2 | 0 | 2 | 1 |
|  | GH114 | 21 | 21 | 12 | 79 | 24 |
|  | GH115 | 1 | 9 | 3 | 4 | 3 |
|  | GH120 | 1 | 0 | 0 | 1 | 0 |
|  | GH124 | 2 | 3 | 0 | 1 | 1 |
|  | GH133 | 1 | 1 | 0 | 1 | 1 |
|  | GH141 | 1 | 1 | 1 | 1 | 0 |
|  | **Glycoside Hydrolases (GHs)** | **262** | **546** | **379** | **473** | **279** |
| **Glycosyl Transferase (GT) Familes** | GT1 | 5 | 5 | 4 | 2 | 4 |
|  | GT2 | 37 | 65 | 30 | 25 | 27 |
|  | GT3 | 2 | 4 | 0 | 2 | 2 |
|  | GT4 | 6 | 10 | 7 | 7 | 7 |
|  | GT6 | 0 | 1 | 0 | 2 | 0 |
|  | GT8 | 4 | 10 | 2 | 5 | 5 |
|  | GT10 | 5 | 4 | 2 | 2 | 2 |
|  | GT11 | 0 | 1 | 1 | 0 | 0 |
|  | GT15 | 1 | 1 | 1 | 1 | 1 |
|  | GT17 | 12 | 10 | 4 | 15 | 6 |
|  | GT20 | 5 | 7 | 6 | 5 | 5 |
|  | GT22 | 4 | 6 | 5 | 3 | 4 |
|  | GT24 | 0 | 1 | 1 | 1 | 1 |
|  | GT31 | 1 | 1 | 0 | 0 | 1 |
|  | GT32 | 2 | 3 | 1 | 1 | 2 |
|  | GT33 | 1 | 1 | 0 | 0 | 1 |
|  | GT34 | 14 | 18 | 15 | 5 | 14 |
|  | GT35 | 1 | 2 | 3 | 1 | 1 |
|  | GT39 | 6 | 12 | 11 | 6 | 7 |
|  | GT41 | 1 | 2 | 1 | 1 | 1 |
|  | GT48 | 1 | 0 | 0 | 0 | 0 |
|  | GT50 | 1 | 1 | 1 | 1 | 1 |
|  | GT58 | 1 | 1 | 1 | 1 | 1 |
|  | GT66 | 1 | 1 | 1 | 1 | 1 |
|  | GT69 | 0 | 0 | 1 | 1 | 0 |
|  | GT71 | 13 | 17 | 8 | 13 | 8 |
|  | GT76 | 1 | 1 | 1 | 1 | 1 |
|  | GT101 | 0 | 3 | 2 | 2 | 1 |
|  | **Glycosyl Transferases (GTs)** | **125** | **188** | **109** | **104** | **104** |
| **Polysaccharide Lyase (PL) Families** | PL1 | 6 | 33 | 19 | 17 | 10 |
|  | PL1_2 | 0 | 0 | 0 | 1 | 0 |
|  | PL1_6 | 0 | 2 | 0 | 0 | 1 |
|  | PL1_7 | 1 | 0 | 4 | 0 | 0 |
|  | PL1_dist | 0 | 0 | 2 | 0 | 0 |
|  | PL3 | 1 | 30 | 8 | 6 | 3 |
|  | PL3_2 | 1 | 29 | 3 | 6 | 3 |
|  | PL4 | 3 | 17 | 3 | 9 | 2 |
|  | PL4_dist | 3 | 17 | 3 | 4 | 2 |
|  | PL9 | 1 | 0 | 0 | 0 | 0 |
|  | PL11 | 0 | 2 | 0 | 0 | 0 |
|  | PL11_2 | 0 | 2 | 0 | 0 | 0 |
|  | **Polysaccharide Lyases (PLs)** | **11** | **82** | **30** | **32** | **15** |

<https://mycocosm.jgi.doe.gov/mycocosm/annotations/browser/cazy/summary;hFIg13>
